# SEX AND TISSUE SPECIFIC ROLES OF MTORC2 IN HEALTHSPAN, METABOLISM, AND SURVIVAL

**DOI:** 10.1093/geroni/igz038.1351

**Published:** 2019-11-08

**Authors:** Dudley W Lamming

**Affiliations:** University of Wisconsin-Madison, Madison, Wisconsin, United States

## Abstract

Genetic and pharmacological inhibition of the mechanistic Target Of Rapamycin Complex 1 (mTORC1) promotes health and longevity in organisms ranging from yeast to mice, and may also have rejuvenative effects in dogs and humans. A potential barrier to the translation of rapamycin-based therapies to the clinic are the side effects of rapamycin, which include metabolic disruption. We and others have demonstrated that many of these side effects are mediated not by inhibition of mTORC1, but by “off-target” inhibition of a second mTOR complex, mTORC2. However, the effect of inhibiting mTORC2 on the healthspan and longevity of mammals has been largely unaddressed. Here, we will discuss our research exploring the contribution of mTORC2 signaling in three different tissues to the healthspan, metabolism, and longevity of mice.

